# Modeling the Adoption of Innovations in the Presence of Geographic and Media Influences

**DOI:** 10.1371/journal.pone.0029528

**Published:** 2012-01-19

**Authors:** Jameson L. Toole, Meeyoung Cha, Marta C. González

**Affiliations:** 1 Engineering Systems Division, Massachusetts Institute of Technology, Cambridge, Massachusetts, United States of America; 2 Graduate School of Culture Technology, Korea Advanced Institute of Science and Technology, Daejeon, Korea; 3 Department of Civil and Environmental Engineering, Massachusetts Institute of Technology, Cambridge, Massachusetts, United States of America; Cajal Institute, Consejo Superior de Investigaciones Científicas, Spain

## Abstract

While there is a large body of work examining the effects of social network structure on innovation adoption, models to date have lacked considerations of real geography or mass media. In this article, we show these features are crucial to making more accurate predictions of a social contagion and technology adoption at a city-to-city scale. Using data from the adoption of the popular micro-blogging platform, Twitter, we present a model of adoption on a network that places friendships in real geographic space and exposes individuals to mass media influence. We show that homophily both among individuals with similar propensities to adopt a technology and geographic location is critical to reproducing features of real spatiotemporal adoption. Furthermore, we estimate that mass media was responsible for increasing Twitter's user base two to four fold. To reflect this strength, we extend traditional contagion models to include an endogenous mass media agent that responds to those adopting an innovation as well as influencing agents to adopt themselves.

## Introduction

In an increasingly digital and connected world, the processes by which information is shared and consumed are changing rapidly. Services and content are now distributed through on-line social networks where the flattening effects of the Internet distort spatial diffusion. These factors are quickly shifting the balance between word-of-mouth and mass media advertisement and along with it, changing the prominent spatiotemporal scales on which spreading occurs. Aiding our ability to characterize and quantify this shift are unprecedented amounts of data elucidating how people communicate with each other and how that communication translates into choices or behaviors such as adopting an innovation or technology.

In this article, we update and unify traditional models of information spread and technology adoption to more accurately reflect the novel economic and social environments in which spreading now occurs. We expand on metapopulation models by embedding social networks in real geography to reflect the spatial distribution of social ties and better understand how local demographics and topology affect contagion. Furthermore, we introduce an endogenous media agent to our network simulation, capturing the role of hyper-influential social forces. Our model is informed by a case study examining the *viral* (as it is colloquially referred) adoption of a social micro-blogging platform, Twitter, where we focus on the accumulation of users in cities across the US over a three year period.

Traditional models of contagion have generally focused on the spread of disease [1] or the diffusion of innovation [2]–[4]. Simple approaches such as the susceptible - infected (SI) model have proven extremely informative, but suffer from overly simple assumptions such as homogeneous mixing of populations. The diffusion of innovations literature has had made use of similar frameworks, such as the Bass model [5], to characterize the adoption of technologies that feature considerable cost and risk. We show, however, that these models perform poorly when applied to goods and services that are nearly cost- or risk-free and demonstrate massive positive externalities like social web applications.

These spreading processes have been placed on networks, revealing how the topology of our social connections aids or hinders outbreaks. The importance of this work continues to grow as the world that becomes increasingly connected by the Internet or cheap and fast travel by cars, trains, and planes [6]–[10]. Few, however, have placed such networks in real geography while preserving individual interactions, thinking carefully about properties such as homophily [11], [12].

More recently, massive popular interest in social networks has lead scholars to recognize the potential of using these platforms as natural experiments on how word-of-mouth information spreading occurs. For example, it has been shown that different types of information, be it political or sports related, follow different patterns as they are shared and consumed by millions of individuals [13], [14]. Some information even takes on a life of its own, evolving into self-sustaining ‘memes’ [15]. In many cases, however, predicting the outcomes of such processes has proven extremely difficult [16].

Social scientists have used similar frameworks to study collective action in the form of binary decisions in order to understand a wide variety of phenomena. Neighborhood segregation [17], riots, technology adoption [18], and standards setting are just a few examples of behavioral contagion studied [19]–[23]. Current events have proven this work increasingly relevant as revolutions and protests are coordinated through these online social networks.

Studies have also explored the many forces influencing the speed and success of information spreading from blogs to traditional news outlets [14], [24], [25]. Research revealed a number of patterns whereby mass media drives conversation on social networks or vice versa. In some instances, it has been found that when advertising effects are controlled for, word-of-mouth diffusion is a negligible force driving adoption [26]. Finally, marketers and retailers have long been examining the various roles of celebrity endorsements as well as spatial diffusion of information about products and services in an attempt to optimize business outcomes [5], [27]–[32].

In this article, we address significant gaps in the above literature. Namely, we show that the geographic distribution of individuals' with differing propensities to adopt (such as early versus late adopters), combined with a preference for friendship with others who share similar tastes and geographic locations, are crucial features to accurately describe micro (at the city level) and macro (at the national level) adoption trends. Furthermore, we propose a model that includes an endogenous mass media that responds to adoption patterns of users while at the same time influencing individuals to adopt an innovation. Based on adoption data from the popular social blogging platform, Twitter, we present a model of contagion to capture salient features. The remainder of this article is organized into three parts: (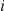) we present analysis of the spatiotemporal adoption of Twitter as a case study, examining the roles of word-of-mouth spreading as well as mass media, (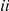) we use insights from the case study to construct a network model and simulate adoption, (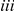) and finally we present and discuss results and important parameters of our model.

## Materials and Methods

### A case study of Twitter

**Figure 1:**
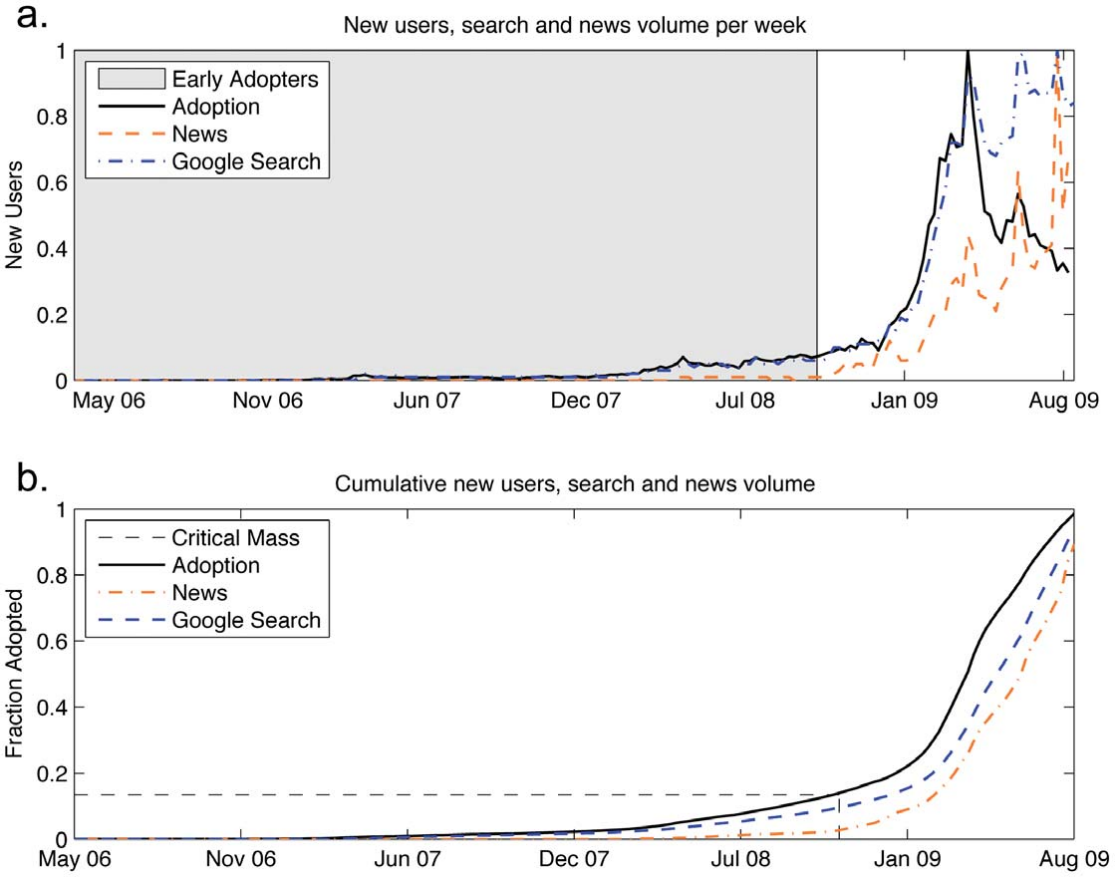
Plots of weekly national adoption. (a.) The number of new U.S. Twitter users is plotted for each week, normalized by the maximum weekly increase during the entire period of data collection. (b.) The cumulative total number of U.S. Twitter users is plotted for for the same time period. Google search and news volumes are normalized such that the maximum value is 1.

As with most complex systems, there are many different scales at which to analyze dynamics. We start at the national level, counting the number of new users that signed up for Twitter within the US each week. Fig. 1 shows time series for both week-to-week user gains as well as the cumulative sum over the first 3.5 years of Twitter's existence. In addition, we have gathered data from Google's Trends and Insights web application measuring weekly search volume news reference volume for the query “Twitter” on Google News. Information on how search and news values are scaled can be found at http://www.google.com/intl/en/trends/about.html.

Following diffusion of innovations literature we label users according to when they adopted relative to all other adopters. For the purposes of this analogy, we make the simplifying assumption that adoption stopped in late 2009. Though this assumption is inaccurate given the subsequent growth of the platform, we do not have data passed this point. Furthermore, our time series suggest that Twitter's growth was slowing down significantly compared to a brief period of extremely fast growth. Those who adopt earlier than 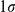 (standard deviation) before the average adoption time are labeled as early adopters. Those adopting between 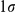 before and the mean adoption time are the early majority, with the late majority and laggards adopting in further deviations past the mean time. For more on the motivations behind this, see Rogers, 1995 [2].

**Figure 2:**
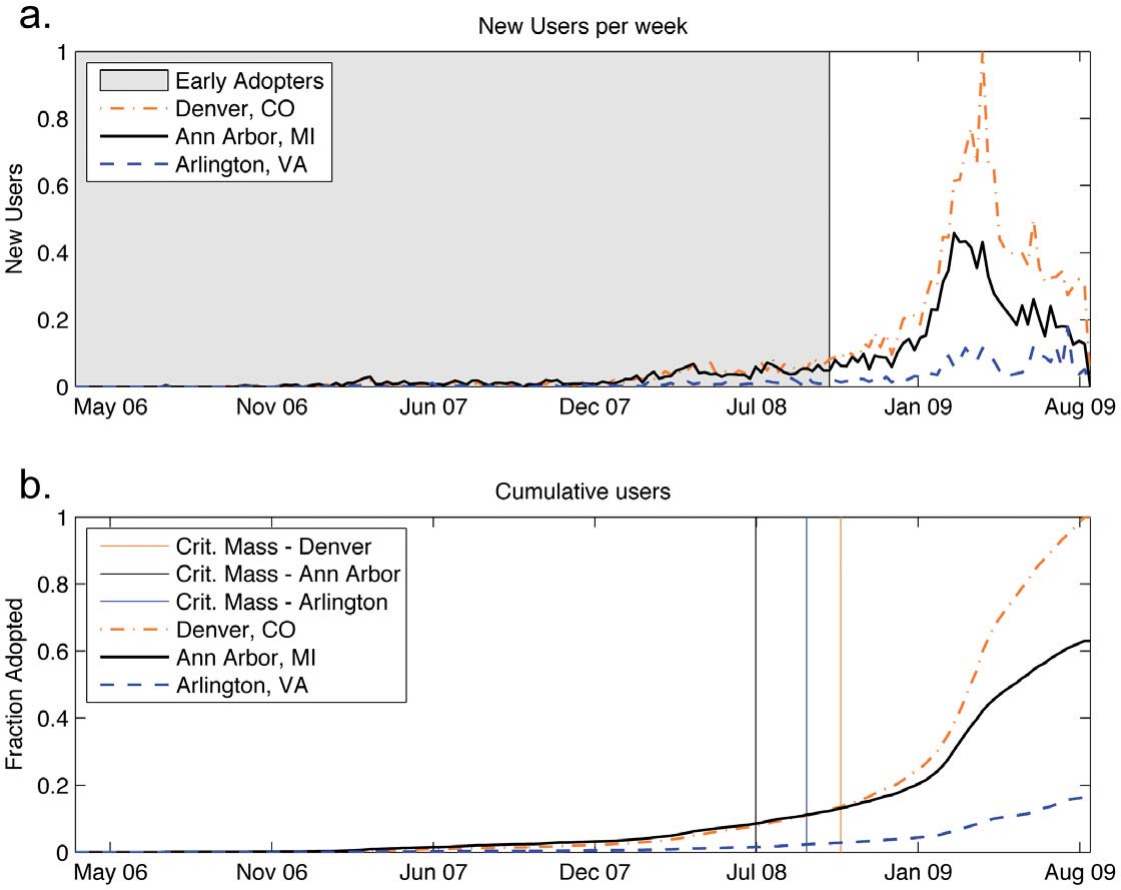
Plots of weekly adoption for select cities. (a.) Time series display the number of new U.S. Twitter users for three separate locations (Ann Arbor, MI, Denver, CO, and Arlington, VA) from mid-March 2006 through late-August 2009, normalized by the largest weekly increase in Denver users. (b.) Shows a plot of the cumulative fraction of each city's user base normalized by the total number of users in Denver, CO.

At the lowest level of spatial resolution available in our data set, we examine the adoption patterns for individual cities. Though we find users in nearly 16,000 cities across the country, many of these locations have only a few users signed up. To ensure enough statistical power, we select only cities with over 1000 users, leaving 408 locations for the remainder of our analysis. Despite this threshold, we still retain data for roughly 70% of all users. Fig. 2 shows three different locations representing a young, early adopting demographic (Ann Arbor, MI), a large metropolitan region consisting mostly of late majority adopters (Denver, CO), and a mixed area (Arlington, VA). While these cities still show the classic S-shaped adoption curves, there are some significant differences such as the large spike in adopters during April, 2009 seen in Denver, CO, but not in Ann Arbor, MI. Later, we argue that these differences are the result of demographics that have different propensities to adopt and respond differently to media influences.

Having labeled adopters relative to the national population, we then measure the composition of each city in terms of the percentage of users who are early adopters, early majority, late majority, or laggards. This step also serves to normalize locations with respect to population. We find, unsurprisingly, that cities with the most early adopters tend to have large universities or are technology centers that tend to attract large numbers of young, tech-savvy persons who are likely to adopt social web applications. Importantly, these locations are not necessarily co-located near each other. College towns all across the country saw early growth of Twitter users despite being very far from major metropolitan areas usually known for driving innovation. Later, we show that the empirical composition of cities and the demographics they represent is critical to reproducing spatiotemporal diffusion patterns.

**Figure 3:**
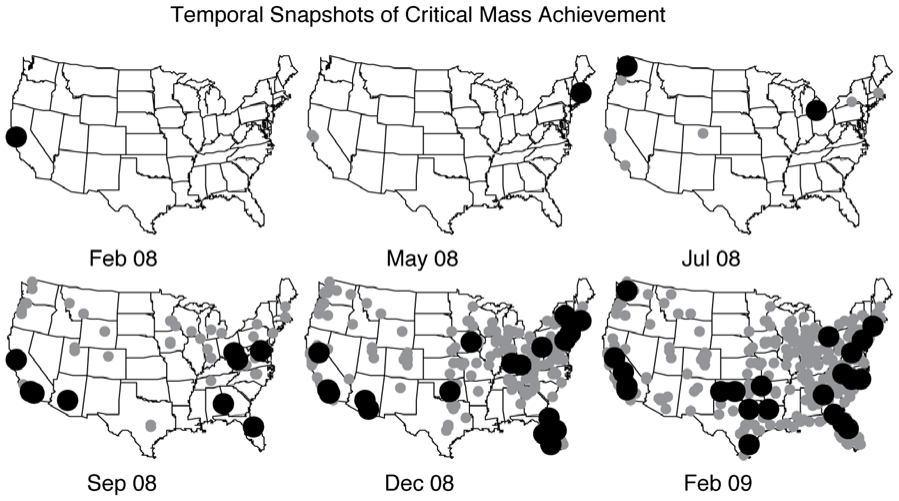
Temporal snapshots of critical mass achievement at locations across the US. For snapshot, the smaller, gray markers indicate locations that have already reached critical mass. The larger, black markers denote locations that achieved critical mass during that week. We note that locations achieving critical mass at very early times are clustered around Twitter's birthplace, San Francisco, CA, suggesting local word-of-mouth diffusion. There are, however, a few locations on the other side of the country, namely the suburbs of Boston, MA that are equally early in adoption, contrasting local diffusion with the flattening effects of the Internet.

We next focus on a key moment for any contagion process, critical mass achievement. Again following conventions from the diffusion of innovations literature, we mark a city as reaching critical mass when 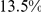 of all eventual users have signed up [3]. Fig. 3 shows a series of snapshots in time indicating when various US cities reach critical mass. These snapshots reveal the diffusion path of Twitter from its birthplace in Silicon Valley, to college towns such as Cambridge, MA, Ann Arbor, MI, or Austin, TX, to metropolitan areas such as Los Angeles, CA, or Denver, CO, then finally to more suburban and rural areas. As noted above, this pattern is non-local in space. Whereas disease can only be transmitted via physical contact, or at least being in the same place at nearly the same time, online innovations are not necessarily constrained by geography and can travel coast-to-coast almost instantaneously. Despite reaching critical mass very early in the San Francisco Bay Area, Twitter did not diffuse spatially up and down the California coast. Instead, it hopped thousands of miles to Cambridge, MA, another highly tech-savvy population. As we shall see later, however, diffusion is not entirely non-local.

Just as individuals users were labeled as early adopters or laggards, cities were also placed into groups according to when they reached critical mass relative to the entire population. Table S1 in the on-line supporting information displays a complete list cities and their classification to illustrate, qualitatively, the type of demographic information that can be inferred from looking at the adoption of web applications. For example, many of the early adopting cities are home to large, public universities whose students are young internet users. Smaller rural towns make up the majority of the lagging cities, with large metropolitan areas falling somewhere in between.

As with any product or service, we expect at least some influence from marketing and advertising, done either explicitly by the creators in the form of advertising and marketing or done implicitly by the users through word-of-mouth. As explained above, we use Google news and search volumes as a proxy for these media influences. Importantly, we note that media coverage (Fig. 1) of Twitter was nearly non-existent during the first two years. The company itself did almost no official advertising. During this time, Google search volume was highly correlated with user growth. After a critical mass of users was reached, media coverage began to increase super-linearly. Many of the spikes in adoption rates were the result of celebrity endorsements such as Oprah's decision to officially sign up on-air during her show on April 17th, 2009 and political events like the Iranian protests in July and August 2009 that sparked debate over the use of social media to coordinate revolution.

Qualitatively, we recognize the media as having an enormous role in driving adoption. We also find that news coverage did not pick up until after the nation had achieved a critical mass of users, suggesting strong endogeneity where media responds to the very adoption it produces. This is much different than the traditional modeling of media [4], [5]. We seek to capture these stylized facts by including a powerful media agent whose coverage both grows with adoption and produces powerful and random shocks, simulating hyper-influentials and major media events.

### Model Introduction

To capture both geographic effects as well as media influence, we introduce our model as follows:

(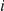) We begin by initializing the agent population and social network. Contagion spreading is simulated by a mechanism resembling the susceptible - infected (SI) model, which is also a special case of the Bass model, widely used in the diffusion of innovations literature. We create a population of 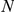 agents and place each agent into one of 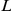 cities, creating city level meta-populations. Each agent can be one of two types, *early adopter* or *regular adopter*. The geographic placement and and agent types are chosen to reflect empirical distributions of real Twitter users as well the composition of cities. Thus, if a city was measured to have 4% of all US Twitter users, 4% of our agents are placed there. Furthermore, of the agents placed in that city, if the composition was measure empirically to be 30% early adopters, 30% of agents will have an early adopter type, with the remainder marked as regular.

Agents are then connected by links to form a social network. The empirical characteristics of links and distances can be set to reflect those measured in on-line social networks. Liben-Nowell et al. [33] show that 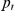, the probability of being connected to someone located a distance 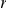 from your city, follows a truncated power-law, 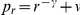, where 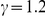 and the probability of connection becomes roughly constant for distances greater than 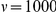 km. We are also able to set the degree distribution and density of the social network to reflect different topologies.

(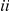) Next, we add dynamics to the simulation. At any given time, each agent can be in one of two states, susceptible (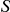) or infected (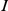). Initial adoption is seeded to a small fraction of agents who are initialized as infected. Spreading is modeled over a series of 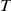 time periods, where the number of agents in each state is tracked (subject to 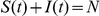). Each time period, all infected agents attempt to infect their neighbors. With probabilities  and 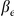, a regular or early adopter, respectively, will heed a recommendation and adopt the technology. We use the ratio, 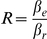 to control differences in propensity to adopt for early versus regular adopters.

These features mimic social dynamics that suggest the pressure to adopt increases as more friends adopt and that more connected people receive greater benefits from adopting social technologies [3]. Some models assume that an individual will adopt an innovation once a specific number [18], [34], [35] or proportion [19] of their contacts have also adopted. Others have found evidence that occupying similar positions in social networks is more predictive of adoption [36]. While we do not attempt to test these hypotheses, Kleinberg has suggested that the dynamics of these adoption schemes are quantitatively similar [37].

(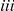) In addition to word-of-mouth spreading, we also incorporate a media agent. This agent can be thought of as an influence in addition to word-of-mouth spreading, similar to the Bass model. Each time period, the media broadcasts its message to adopt a technology, and each agent flips a coin determining if adoption occurs. The media transmission probability is given by, Pr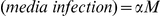, where 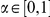 is a model parameter, and 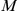 is the endogenous media volume. Media volume itself is determined as a function of the number of previously infected agents, 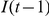, and a random term 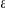 such that 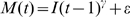. For convenience, we normalize the media so that, 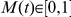. The parameter, 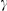, reflects the super-linear growth displayed in Google news media volume. Finally, we set the size of random shocks 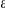 to be on the order of 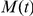, reflecting stylized features seen in Google News volume data.

In essence, the amount of media exposure an innovation is given depends explicitly on the number of people who have adopted it as well as a random error term. Just because the media is reporting on a new product, however, does not mean a consumer will adopt it. To model this, we have included the parameter 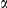, which adjusts how receptive agents are to the media. The probability that any given agent will adopt due to the medias influence, 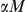, is then given by the product of how much the media is reporting and how closely an individual is listening.

## Results

### Replicating standard SI model

We first present results for parameter settings that reduce our simulation to the traditional SI model. We set 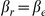 (leaving only one type of agent), 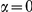 (removing the media), and populate each of 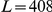 cities uniformly with 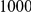 agents for a total population of 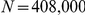. We initialize the network to have a completely random spatial distribution of links so as to remove any geographic bias in friendship and simulate homogeneous mixing in the population. We choose a Poisson degree distribution because the qualitative structure of the adoption network is more selective than a scale free structure found in measurements of all connections in online social networks [25], [38], [39]. For example, Leskovec et al. [25] found that individuals who recommended a product to tens or even hundreds of contacts influenced no more purchases on average than those who sent recommendations to just a few friends.

Thus, we expect the number of people who can influence a person to adopt a technology is smaller than the number of acquaintances they have and the distribution is not likely to be long tailed. Scaling these numbers to fit our simulation size we choose a reasonable average degree of 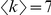.

**Figure 4:**
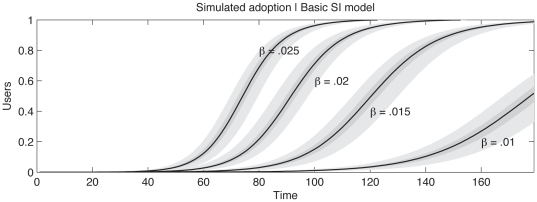
Verification of the basic SI model. Four different transmission rates 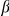 are displayed, each run 500 times and averaged. The bands surrounding the average value are bounds containing 75%, and 95% of simulation runs.

Fig. 4 displays the simulated number of adopters per week for a variety of values for 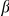. The simulation was run 500 times for each parameter configuration. The bands surrounding the average represent ranges between which 75% and 95% of simulations fell. In this simple form of the model, it is not possible to reproduce the empirical shape of the cumulative adoption curve seen in the Twitter case study suggesting more complicated dynamics are required to accurately predict the adoption of these technologies.

Next, we add more diverse geography to the model in the form of city populations, geographically distributed friendships, and early adopters that are three times as likely to adopt when than regular adopters (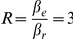). To understand how these additions affect adoption at the local level, we first examine the importance of network structure in the presence of two agent types.

Our analysis shows that homophily based solely on agent type (i.e. early versus late adopter) is not enough to reproduce the observed trends in the spatiotemporal diffusion of information. A very specific type and strength of homophily must be present to ensure that the early adopters are connected to each other, forming a giant component in the early adopter sub-network, and not leaving members of their type isolated by regular adopters. To introduce these different types of homophily into the network, we simulate two types of networks, homogenous mixing and spatially embedded networks, and also vary the fraction of similarly typed neighbors each agent prefers. In order to form giant component of early adopters, we find that not only must agents prefer friendships with other agents of similar type (homophily by type), but they must also prefer friendships with those closer to them geographically, forming a *spatial social network*. Spatially embedded friendships are selected as a function of distance with probability,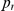, of selecting a friend who is a geographic distance 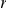 away is described in the previous sections [33].

**Figure 5:**
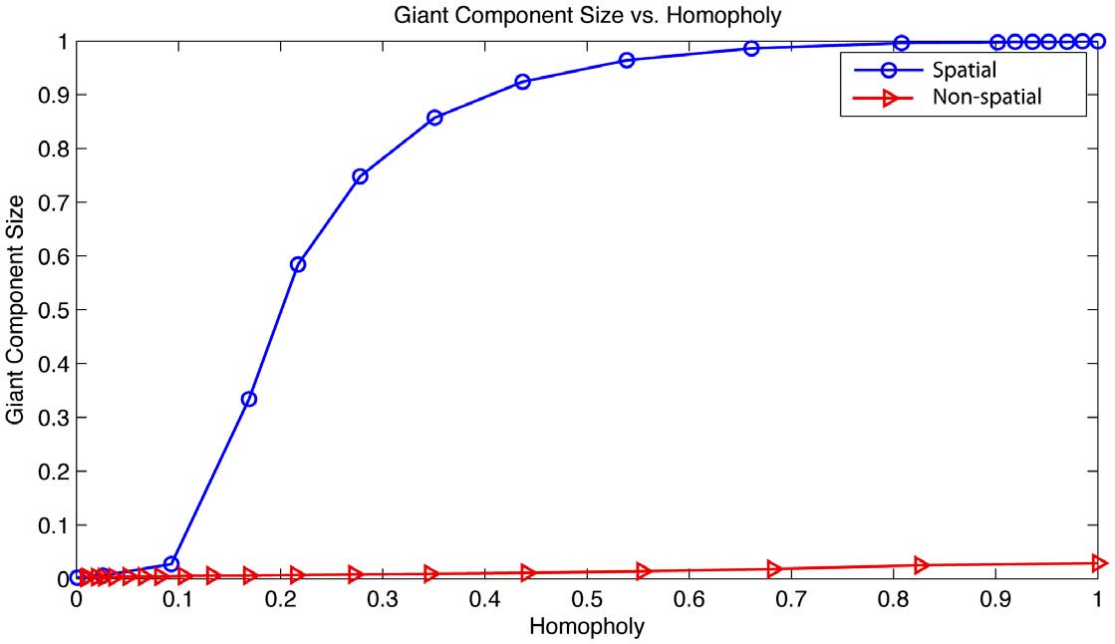
The size of the giant component plotted against homophily. Two configurations are shown, one in which the social network is explicitly spatial, the other ignoring geography of nodes. The figure illustrates that preference for friendship with similar agents is not enough to connect early adopters in a giant component and that spatial friendships are produce this structure.

Fig. 5 plots the size of the giant component of early adopters produced at a given level of homophily measured among early adopters for networks either spatially embedded or not. Here we define homophily as the average fraction of an early adopter's friends who are also early adopters. These estimates were obtained by creating and consolidating results over 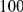 networks, each with 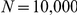 nodes and a given level of homophily, then measuring the size of the giant component. For the remainder of this paper, all configurations labeled *spatial network* can be assumed to have a giant component containing over 95% of all early adopters.

**Figure 6:**
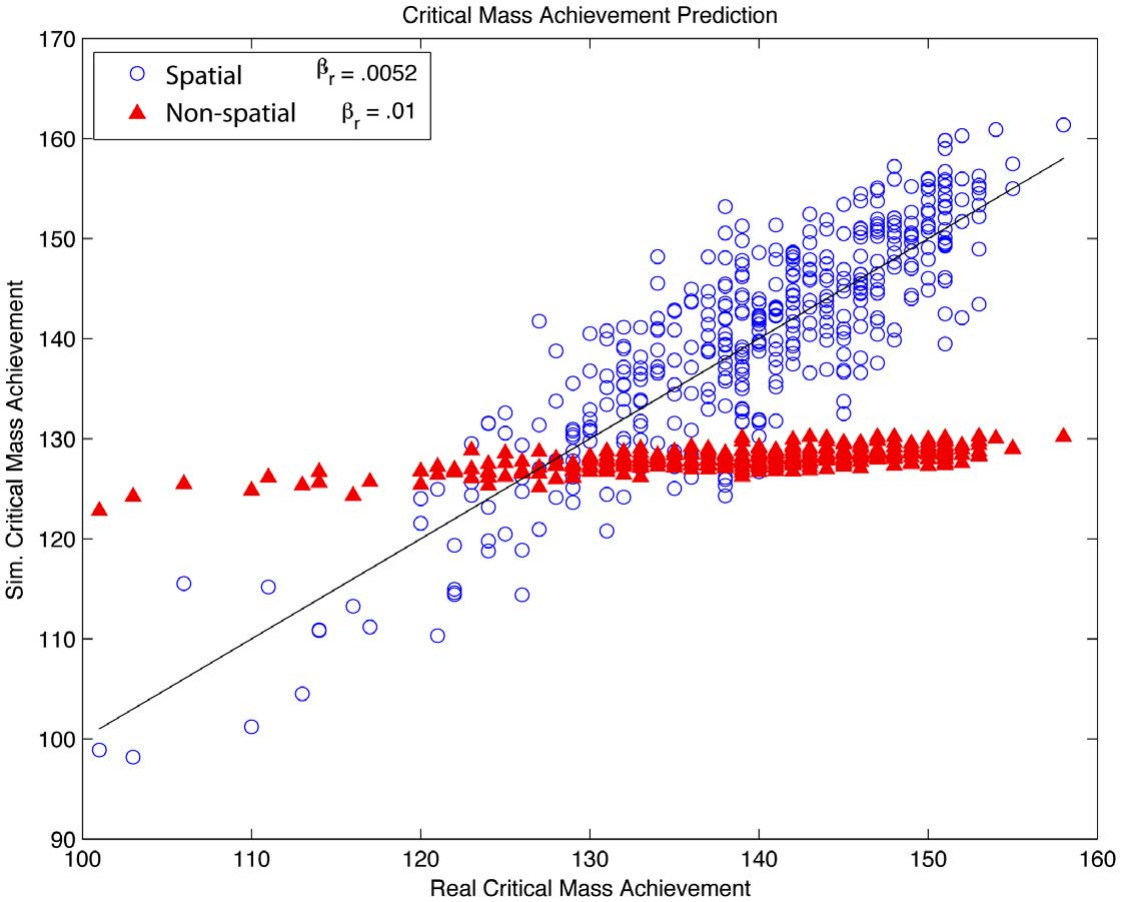
Simulated critical mass achievement times are compared to times measured from Twitter data. We find spatially embedded friendships are necessary to reproduce the inter-city spread of Twitter.

**Figure 7:**
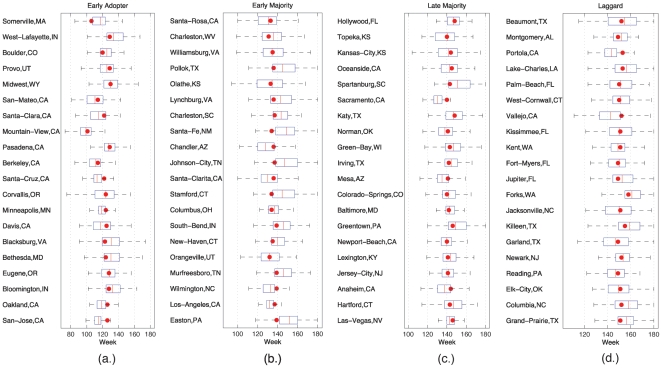
Simulation results are compared to actual critical mass achievement times for different subsets of locations. Borrowing from the diffusion of innovations literature, we use four groups (a.) Early adopting, (b.) Early Majority, (c.) Late Majority, (d.) Laggards. We are able to reliably predict adoption times for cities in each category.

To see how this giant component of early adopters affects adoption, Fig. 6 compares the predicted and actual times of critical mass achievement both with and without spatial friendships. In the absence of a giant component, nearly all cities peak at the same time. When spatially embedded friendships are introduced such that a giant component of early adopters is formed, we are able to simulate city level Twitter adoption, while preserving national trends. Though global cumulative adoption can be reproduced without the spatial social network, adoption cannot is not geographically resolved to the city level. Embedding the social network in real space, however, makes it possible to accurately simulate the critical mass achievement times in most cities. Fig. 7 shows these simulated times when compared to times empirically measured in data. We have divided specific cities into four groups based on when they reached critical mass relative to all locations. For selected cities, simulation quartiles are plotted along with actual peak times. In the on-line supporting information we provide data files containing the composition and adoption times of different cities, with the goal of facilitating future studies of other hypothesis and types of adoption.

### Media Influence

**Figure 8:**
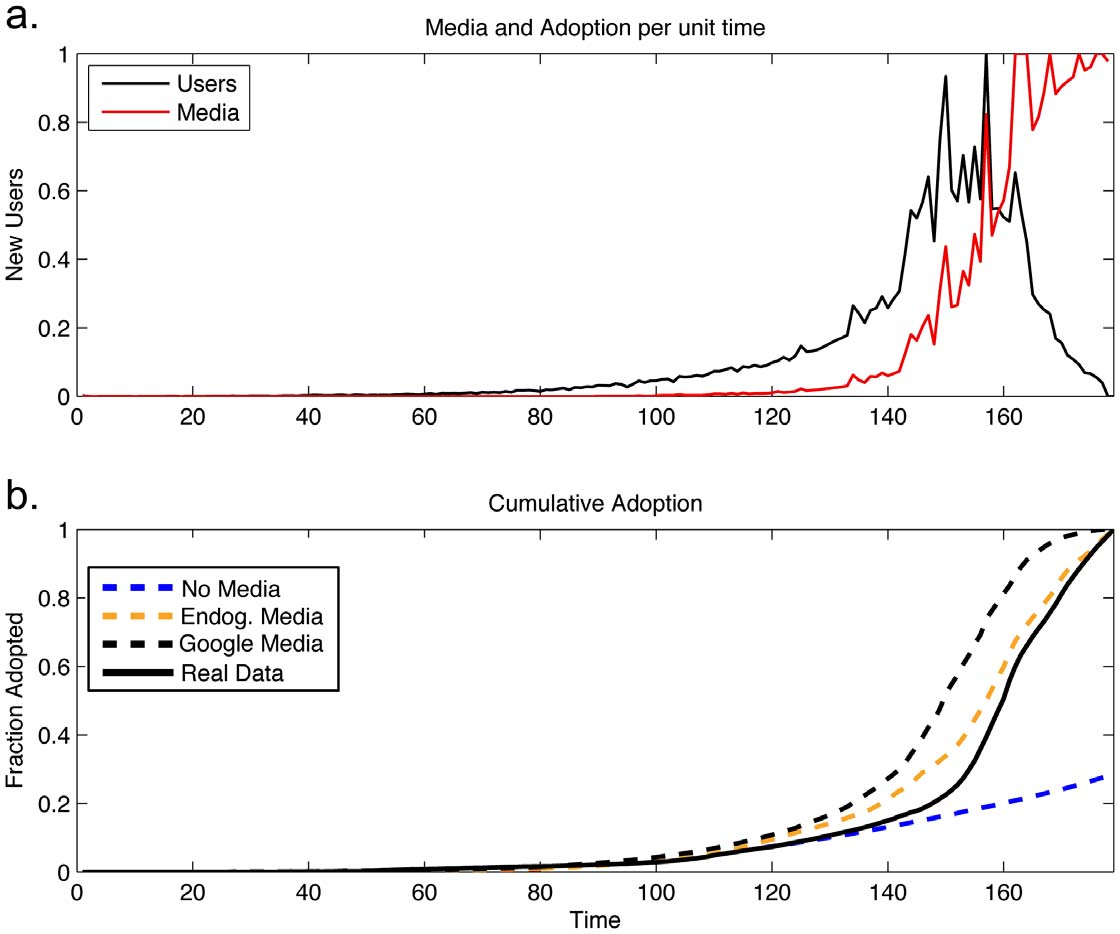
Simulated adoption treating the media as endogenous and increasing with the number of adopters. (a.) Shows simulated new users per week (normalized to the maximum over the period) as well as normalized media volume each week. (b.) A comparison of all model scenarios is shown. Traditional models, models which do not include media influence are capable of predicting adoption in early periods, but dramatically underestimate total adoption. Including endogenous media effects allows us to make adoption predictions that more closely resemble real data.

Fig. 8 compares predictions of national adoption with the above model conditions. Examining news volume as collected by Google, we note that purely word-of-mouth simulations start diverging from reality around week 120 after launch. This is just around the time when mass media begins to report on the web application. Because of this sharp transition, we can measure the relative strength of word of mouth spreading versus mass media influence.

Predicting when the individual media events like celebrity endorsements will occur is beyond the scope of this work. We can, however, simulate adoptions in the presence of empirical news volume from Google's database. In order to achieve the national adoption pattern similar to that seen in real data, we find that agents must be highly susceptible to media influence, with the parameter 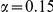. Contrasting aggregate adoption predictions both with and without media influence suggests that the mass media was responsible for at least half of the newly joined Twitter's users, most of whom adopted in later stages. Coupled with our early results showing the importance of homophily and geography during the early stages of spread, our model paints a much more complete picture of adoption, capable of reproducing both aggregate and local trends in space and time.

Next, we expand our model to treat news volume as endogenous such that adoption may be simulated without requiring external empirical data on media influence. We introduce an endogenous mass media, implemented as described above in step *iii*. of our model introduction. Reflecting trends seen in the real data, the growth of media volume is super-linear with respect to adopters and random spikes in media coverage are introduced to reflect discrete and unpredictable media events. For these simulations, we found an exponent of media growth with respect to adopters, 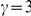, produced reasonable fits to real data. Fig. 8 displays simulation results for various model settings described in this paper. While spatial friendship networks are able to reproduce early adoption trends, real data quickly diverges in later times. Introducing an endogenous mass media agent which grows super-linearly in the number of current adopters produces, along with random media spikes, produces much more accurate adoption trends and reflects features seen real media coverage.

## Discussion

In light of a globalized world with near universal access to the Internet, previous models of adoption fail to characterize the interplay of media and word of mouth. In this article, we have presented descriptive statistics of the spatiotemporal adoption of a web application and proposed a model of technology adoption or, more generally, social contagion, to replicate features seen in data from city to national scales. For early stages, when spreading occurs primarily through word-of-mouth, we find that adoption is strongly correlated with traditional demographic covariates. Early adopting cities tend to be those with large, young, and tech-savvy populations. Media influences during later stages, however, were found to be very strong, accounting for a two to four fold increase in the number of people who adopted. This finding is consistent with earlier work that suggests advertising campaigns are enough to confound any word-of-mouth spreading [26].

Our model extends previous work in two important ways. First, we demonstrate that spatial social networks are crucial to reproducing the dynamics of adoption at a city scale. Secondly, the media features of the model reflect empirical observations that the news volume reacts to the number of adopters with a super-linear trend after a product has reached a critical mass and with random shocks emanating from super-influential people like celebrities or major media events like massive demonstrations.

These results suggest that our model is capable of replicating both micro (at the city level) and macro (at the national level) adoption phenomena and may provide substantial improvement over existing frameworks such as the SI or Bass models. We do, however, urge some caution in the interpretation of our results. Because our simulation relies upon the fraction of a city denoted as early adopters and this fraction was measured empirically from data, the model may be sensitive to errors in this measurement. While our empirical results are intuitive, for example finding that Silicon Valley and college towns have the most early adopters of a viral web application, they may not hold for other products such as durable goods. Our model is best applied to goods and services that are very low cost, very easy to tell someone about, and display large positive externalities.

We hope it inspires future work in the area. Specifically, it would be interesting to compare and contrast the spatial diffusion of web apps such as Twitter, with more tangible products such as gadgets, medicine, or cars. For example, it may be possible to use the composition of the cities as characterized by the adoption of Twitter to predict or even try to accelerate the adoption of other related kinds of technological innovations. To facilitate further research in this area, we have provided a readme and data file, Text S1 and Dataset S1, containing city level compositions as well as time series data in the online supporting information as well as our web page, http://humnet.scripts.mit.edu/wordpress/2011/06/13/project-modeling-the-diffusion-of-social-contagion/. This work also represents advances in models of spreading in networks where the roll of demographics, i.e. node attributes, as well as geography is critical for future predictions. These insights may be particularly useful in modeling opinion spreading such as in elections and collective action.

## Supporting Information

Table S1 **Sample cities within each classification (early adopting, late majority, etc.).** Early adopting cities tend to be college towns or have large populations of young, tech-savy users such as Mountain View, CA, while larger metropolitan areas adopted closer to the mean, followed by more rural and remote locations.
(PDF)

Text S1 **A readme file containing information on the files included in Dataset S1.**
(TXT)

Dataset S1 **A dataset containing city level composition data as well as time series of weekly adoption.** For each of the 408 cities used as input in our model, we have included data such as the percentage of early adopters, the total population of Twitter users, and weekly time series of new users.
(XLS)
