# Supplementary material for: Modeling the Adoption of Innovations in the Presence of Geographic and Media Influences
Source: PLoS One. 2012 Jan 19;7(1):e29528. doi: 10.1371/journal.pone.0029528 (PMC3261844; doi:10.1371/journal.pone.0029528)
Supplement: Table S1 — Sample cities within each classification (early adopting, late majority, etc.). Early adopting cities tend to be college towns or have large populations of young, tech-savy users such as Mountain View, CA, while larger metropolitan areas adopted closer to the mean, followed by more rural and remote locations. (PDF) [file pone.0029528.s001.pdf]

Table S1: Sample cities within each classification (early adopting, late majority, etc.) Early adopting cities tend to be college towns or have large populations of young, tech-savy users such as Mountain View, CA, while larger metropolitan areas adopted closer to the mean, followed by more rural and remote locations.

**Early Adopter**	**Early Majority**	**Late Majority**	**Laggard**
**60**	**125**	**157**	**66**
Ames,IA	Akron,OH	Abilene,TX	Amarillo,TX
Ann-Arbor,MI	Albany,NY	Albright,WV	Beaumont,TX
Arlington,VA	Alexandria,VA	Albuquerque,NM	Bronx,NY
Austin,TX	Alpharetta,GA	Allentown,PA	Cheshire,CT
Beaverton,OR	Amsouth-Bank,TN	Anaheim,CA	Chesterland,OH
Bellevue,WA	Anchorage,AK	Arlington,TX	Clarksville,TN
Bellingham,WA	Anderson,SC	Augusta,GA	Cleveland,GA
Berkeley,CA	Annapolis,MD	Aurora,CO	College-Park,GA
Bethesda,MD	Appleton,WI	Bailey,CO	Columbia,NC
Blacksburg,VA	Asheville,NC	Bakersfield,CA	Columbus,GA
Bloomington,IN	Athens,OH	Baltimore,MD	Corpus-Christi,TX
Bluefield,VA	Athens-Clarke-County,GA	Baton-Rouge,LA	Detroit,MI
Boston,MA	Atlanta,GA	Bayville,NJ	El-Paso,TX
Boulder,CO	Auburn,AL	Bethlehem,PA	Elk-City,OK
Bozeman,Mt	Bend,OR	Beverly-Hills,CA	England,AR
Cambridge,MA	Boca-Raton,FL	Billings,Mt	Fayetteville,NC
Cary,NC	Boise,ID	Biloxi,MS	Flint,MI
Chapel-Hill,NC	Brooklyn,NY	Birmingham,AL	Fort-Myers,FL
Charlottesville,VA	Burbank,CA	Bowling-Green,KY	Garland,TX
Corvallis,OR	Carlsbad,CA	Bradenton,FL	Grand-Prairie,TX
Davis,CA	Cedar-Rapids,IA	Buffalo,NY	Hamilton,OH
Des-Moines,IA	Champaign,IL	Canton,OH	Hattiesburg,MS
Eugene,OR	Chandler,AZ	Cape-Coral,FL	Hebron,KY
Evanston,IL	Charleston,SC	Charlotte,NC	Jackson,MS
Fairfax,VA	Charleston,WV	Chesapeake,VA	Jacksonville,NC
Franklin,TN	Chattanooga,TN	Cincinnati,OH	Jeffersonton,VA
Grand-Rapids,MI	Chicago,IL	Clearwater,FL	Jupiter,FL
Hoboken,NJ	Chico,CA	College-Station,TX	Kent,WA
Ithaca-College,NY	Cleveland,OH	Colorado-Springs,CO	Killeen,TX
Livermore,CA	Columbia,MO	Columbia,SC	Kissimmee,FL
Madison,WI	Columbus,OH	Dallas,TX	Lake-Charles,LA
Midwest,WY	Computer-Com-of-Amer,DE	Dayton,OH	Laredo,TX
Minneapolis,MN	Conway,AR	Decatur,GA	Lexington,OK
Missoula,Mt	Coral-Springs,FL	Duluth,MN	Long-Beach,CA
Mountain-View,CA	Costa-Mesa,CA	Durango,CO	Lubbock,TX
Oakland,CA	Denton,TX	Elk-Grove,CA	McAllen,TX
Palo-Alto,CA	Denver,CO	Evansville,IN	Miami,FL
Pasadena,CA	Durham,NC	Everett,WA	Mobile,AL
Portland,ME	East-Lansing,MI	Fayetteville,AR	Modesto,CA
Portland,OR	Easton,PA	Fort-Lauderdale,FL	Montgomery,AL
Provo,UT	Fort-Collins,CO	Fort-Wayne,IN	New-Ringgold,PA
Reston,VA	Frederick,MD	Fort-Worth,TX	Newark,NJ
Rochester,MN	Fredericksburg,VA	Fresno,CA	Newfoundland,PA
Round-Rock,TX	Fremont,CA	Gilbert,AZ	Newport-News,VA
Salt-Lake-City,UT	Frisco,TX	Glendale,AZ	Nokesville,VA
San-Francisco,CA	Fullerton,CA	Glendale,CA	Ocala,FL
San-Jose,CA	Gainesville,FL	Greeley,CO	Palm-Beach,FL
San-Mateo,CA	Greenville,SC	Green-Bay,WI	Palmdale,CA
Santa-Barbara,CA	Gresham,OR	Greensboro,NC	Philippi,WV
Santa-Clara,CA	Harrisburg,PA	Greentown,PA	Portola,CA
Santa-Cruz,CA	Henderson,NV	Greenville,NC	Prosper,TX

Santa-Monica,CA
Seattle,WA
Silver-Spring,MD
Somerville,MA
St-Paul,MN
State-College,PA
Sunnyvale,CA
Venice,CA
West-Lafayette,IN

Honolulu,HI
Huntsville,AL
Iowa-City,IA
Irvine,CA
Johnson-City,TN
Kalamazoo,MI
Kansas-City,MO
Knoxville,TN
Lansing,MI
Lawrence,KS
Lawrenceville,GA
Leavenworth-Lake-Wenatchee,WA
Lincoln,NE
Littleton,CO
Los-Angeles,CA
Lynchburg,VA
Manchester,NH
Manhattan,KS
Marietta,GA
Miami-Beach,FL
Milwaukee,WI
Muncie,IN
Murfreesboro,TN
Napa,CA
Naperville,IL
New-Haven,CT
Newark,IL
North-Hollywood,CA
Olathe,KS
Olympia,WA
Omaha,NE
Orange,CA
Orangeville,UT
Orlando,FL
Overland-Park,KS
Petaluma,CA
Philadelphia,PA
Phoenix,AZ
Pittsburgh,PA
Plano,TX
Pollok,TX
Raleigh,NC
Redondo-Beach,CA
Reno,NV
Richmond,VA
Rochester,NY
Salem,OR
San-Buenaventura-(Ventura),CA
San-Diego,CA
San-Luis-Obispo,CA
San-Marcos,TX
Santa-Clarita,CA
Santa-Fe,NM
Santa-Rosa,CA
Sarasota,FL
Scottsdale,AZ

Hartford,CT
Hayward,CA
Heart-Butte,Mt
Hollywood,FL
Holtsville,NY
Hope,NY
Houston,TX
Huntington,WV
Huntington-Beach,CA
Indianapolis,IN
Irving,TX
Jacksonville,FL
Jersey-City,NJ
Jersey-Shore,PA
Joliet,IL
Kansas-Bank-Amer,KS
Kansas-City,KS
Katy,TX
Kennesaw,GA
Kula,HI
Lafayette,IN
Lafayette,LA
Laguna-Beach,CA
Lakeland,FL
Lancaster,PA
Las-Cruces,NM
Las-Vegas,NV
Lexington,KY
Little-Rock,AR
Louisville,KY
Loveland,OH
Macon,GA
Malibu,CA
Marion,IN
McKinney,TX
Melbourne,FL
Melbourne,IA
Memphis,TN
Mesa,AZ
Midland,TX
Millersville,MD
Monongahela,PA
Morgantown,WV
Murrieta,CA
Myrtle-Beach,SC
Naples,FL
New-Brunswick,NJ
New-Orleans,LA
New-York,NY
Newark,DE
Newport-Beach,CA
Norman,OK
Oceanside,CA
Oklahoma-City,OK
Orange,TX
Palm-Springs,CA

Queens,NY
Reading,PA
Shreveport,LA
Stilwell,OK
Stockton,CA
Upper-Marlboro,MD
Valdosta,GA
Vallejo,CA
Visalia,CA
West-Cornwall,CT
Whittier,CA
Wilmington,DE
Winston-Salem,NC
Yonkers,NY

Sioux-Falls,SD
South-Bend,IN
Spokane,WA
Springfield,IL
St-Louis,MO
Stamford,CT
Stillwater,OK
Tempe,AZ
Thousand-Oaks,CA
Tulsa,OK
Tustin,CA
Vancouver,WA
Washington,DC
West-Hollywood,CA
Williamsburg,VA
Wilmington,NC
Winter-Park,FL
Woonsocket,RI

Panama-City,FL
Pensacola,FL
Peoria,AZ
Peoria,IL
Piatt,PA
Pinckney,MI
Providence,RI
Puyallup,WA
Rancho-Cucamonga,CA
Redding,CA
Riverside,CA
Roanoke,VA
Rockford,IL
Roseville,CA
Sacramento,CA
San-Antonio,TX
San-Bernardino,CA
San-Clemente,CA
Savannah,GA
Scranton,PA
Siloam-Springs,AR
Simi-Valley,CA
Spartanburg,SC
Springfield,MO
St-Augustine,FL
St-Petersburg,FL
St-Stephen,SC
Staten-Island,NY
Sugar-Land,TX
Surprise,AZ
Syracuse,NY
Tacoma,WA
Tallahassee,FL
Tampa,FL
Temecula,CA
Toledo,OH
Topeka,KS
Torrance,CA
Traverse-City,MI
Tucson,AZ
Tuscaloosa,AL
Tyler,TX
Virginia-Beach,VA
Waco,TX
West-Chester,PA
West-Palm-Beach,FL
Winchester,VA
Woodbridge,VA
Worcester,MA
Young,AZ
